# Untangling the perception of value in value-based healthcare – an interview study

**DOI:** 10.1108/LHS-07-2023-0051

**Published:** 2024-04-16

**Authors:** Axel Wolf, Annette Erichsen Andersson, Ewa Wikström, Fredrik Bååthe

**Affiliations:** Institute of Health Care Sciences, Sahlgrenska Academy, University of Gothenburg, Gothenburg, Sweden and Centre for Person-Centred Care (GPCC), University of Gothenburg, Gothenburg, Sweden; Institute of Health Care Sciences, Sahlgrenska Academy, University of Gothenburg, Gothenburg, Sweden and Department of Orthopedics, Sahlgrenska University Hospital, Gothenburg, Sweden; Department of Business Administration, School of Business, Economics and Law, University of Gothenburg, Gothenburg, Sweden, and; Institute for Studies of the Medical Profession, LEFO, Oslo, Norway; Institute of Stress Medicine at Region Västra Götaland, Gothenburg, Sweden and Institute of Health Care Sciences, Sahlgrenska Academy, University of Gothenburg, Gothenburg, Sweden

**Keywords:** Professional logics, Leaders, Institutional logics, Value-based health care, Patient-reported experience, Patient-centred care, Person-centred care

## Abstract

**Purpose:**

Value-based health care (VBHC) argues that health-care needs to re-focus to maximise value creation, defining value as the quota when dividing the outcomes important for the patient, by the cost for health care to deliver such outcomes. This study aims to explore the perception of value among different stakeholders involved in the process of implementing VBHC at a Swedish hospital to support leaders to be more efficient and effective when developing health care.

**Design/methodology/approach:**

Participants comprised 19 clinicians and non-clinicians involved in the implementation of VBHC. Semi-structured interviews were conducted and content analysis was performed.

**Findings:**

The clinicians described value as a dynamic concept, dependent on the patient and the clinical setting, stating that improving outcomes was more important than containing costs. The value for non-clinicians appeared more driven by the interplay between the outcome and the cost. Non-clinicians related VBHC to a strategic framework for governance or for monitoring different continuous improvement processes, while clinicians appreciated VBHC, as they perceived its introduction as an opportunity to focus more on outcomes for patients and less on cost containment.

**Originality/value:**

There is variation in how clinicians and non-clinicians perceive the key concept of value when implementing VBHC. Clinicians focus on increasing treatment efficacy and improving medical outcomes but have a limited focus on cost and what patients consider most valuable. If the concept of value is defined primarily by clinicians’ own assumptions, there is a clear risk that the foundational premise of VBHC, to understand what outcomes patients value in their specific situation in relation to the cost to produce such outcome, will fail. Health-care leaders need to ensure that patients and the non-clinicians’ perception of value, is integrated with the clinical perception, if VBHC is to deliver on its promise.

## Introduction

Value-based health care (VBHC) has been proposed as a paradigm shift in health-care management in response to rising health-care costs and quality deficiencies (Porter *et al.*, 2016; Porter and Teisberg, 2006). According to Porter and Teisberg (2006), value is the result of patients’ experienced outcome from the care that was provided, divided by the cost to deliver that outcome, and is referred to as “the value quota”. Consequently, the focus of VBHC is increasing the value quota by delivering better health outcomes as experienced and defined by the patient (a complex mix not only of survival but also of how the patient feels, functions and recovers) while striving for cost containment or cost reduction (Porter, 2009). The challenge of using the concept of “value” is that it means different things to different people in different contexts and episodes. Value is a construct that comprises both attitudes and experiences and is affected by the context and the situation (Sánchez-Fernández and Iniesta-Bonillo, 2006; Benson *et al.*, 2003) and by one’s identity, regardless of whether that identity is of a clinician (e.g. professional care provider), a non-clinician (manager, finance) or a patient. In modern health-care management, concepts such as quality-adjusted life-years within health economics, which combine life years gained with the patient’s perception of quality of life, conceptualise value as not only life-prolonging treatment outcomes, but also as the patient living a fulfilling life. According to the Institute of Medicine, one of the priorities for health care is to start asking patients “what matters to you?” rather than “what is the matter with you?”(Kebede, 2016).

Scholars argue that value is “satisfaction of a want”, that is, the product of the trade-off in a normative picture of the product/service acquired (DeSouza, 1992). Woodruff and Gardial (1996) suggested that value includes a relation between the “want” of a service/product, the recipient and the actual perception from using the service/product. While value could be discussed as a universal perception that combines attitudes, traditions and symbols, it may not be directly associated with the outcome (Fredriksson *et al.*, 2015). Therefore, the concept of value could be understood differently between different clinicians (Erichsen *et al.*, 2015), as well as between clinicians and non-clinicians (in this article defined as hospital personnel not directly interacting with patients).

The perceptions, assumptions and attitudes of groups of people, such as clinicians and non-clinicians, form a shared common logic that together with symbols and rituals is institutionalised internally as well as externally by its members and the surrounding context (Lindberg, 2014). Professional logics are legitimised and brought to the surface by material things and symbols that provide a normative framework, or “rules of legitimacy” (Lindberg, 2014). Therefore, different logics are established via action and practice. Research has shown that different logics can simultaneously compete (Nilsson and Sandoff, 2017) and co-exist within an organisation, not least in institutional environments with strong professional groups, such as in health care (Arman *et al.*, 2014; Wikström and Dellve, 2009). Well-established institutions with strong professional groups, such as hospitals, can also have a set of shared assumptions and symbols across professional logics that define institutional logic (Scott, 2008a, 2008b).

### Health-care leadership

Avolio *et al.* (2009) concludes that leadership in health-care organisations is characterised by complex interactions between “the co-existence of an emerging, government-prescribed, results-oriented approach to leadership (the new institution) with a more traditional professional value-based approach (the old institution)” (p. 664). The hospital is an institution that comprises complex interplay between different logics. According to Glouberman and Mintzberg (2001), there are four foundational logics in the hospital context; community (politicians and policymakers), control (managers and administrators), cure (biomedical expertise, physicians) and care (nursing and other health professionals). There is a great divide among those who work clinically (physicians nurses and other health professionals) from those who do not (policymakers and managers). Each logic is driven by its own way of understanding rules and regulations, and this explains why discrepancies and frustration can occur when different logics with limited shared understandings need to co-operate in creating good quality patient care (Andersson, 2015).

Earlier studies on leadership in health-care organisations have suggested that distributed leadership is an approach to handle the influence of multiple institutional logics in the external context (cf. Currie and Lockett, 2011; Currie *et al.*, 2009a, 2009b). Another example is Currie *et al.* (2011) which highlight the potential of distributed leadership and “managed partnerships” in health-care organisations. It is concluded that distributed leadership in health-care organisations supports the enactment of inherent bureaucracy, power differentials between network participants and a strong centralised performance management policy regime. Accordingly, it is suggested in leadership research that the health-care organisations require a certain type of leadership, and one could argue that the concept of VBHC (Porter and Teisberg (2006) is a way towards integrating “the old institution with the new institution” by granting patients the power to define what is the value of different health-care outcomes.

The implementation of VBHC challenges both the institutional and professional logics of different groups in the hospital. It is a concept impacting both management and professionals, which means that the success of implementing VBHC in a hospital is likely to be affected by how well the concept is explicitly focused on providing enhanced value for patients, by some researchers considered the one dimension that can unite the different logics in the hospital context (Nilsson *et al.*, 2017). A recent scoping review by van Staalduinen *et al.* (2022) indicates that there is a lack of understanding how VBHC is conceptualised and implemented in hospitals. It reveals that most hospitals adopt only a few components of VBHC, primarily focusing on measuring outcomes and costs, with limited examination of the effects and strategies of VBHC implementation. The review underscores the need for a unified conceptualisation of VBHC and concludes with a call for an interdisciplinary approach integrating insights from both health care and the broader management research community, to further advance this field. This study responds to that call for research and will explore the perception of value among different groups within the health-care setting, as implementing VBHC is likely to result in confrontations between different perspectives and logics within the health-care organisation.

### Aim

To explore the perception of value among stakeholders with different professional logic (clinicians and non-clinicians) during the implementation of VBHC.

## Method

### Design and setting

This study used a qualitative design using interviews with different stakeholders during the implementation of VBHC at a large university hospital in Sweden. This methodology aligns well with our objective, as it delves into understanding the meanings individuals assign to their experiences within their natural contexts, as emphasised by Malterud (2001, 2014). Interviews were selected as our method for gathering empirical data, as they provide a direct avenue to comprehend the world from the perspectives of others, an approach advocated by Kvale and Brinkman (2009). The choice of a qualitative approach, particularly through interviews, is apt for capturing the nuanced views of various stakeholders involved in VBHC implementation. These stakeholders, who bring different professional logics to the table, offer rich insights into the concept of value as perceived in their unique roles and experiences.

### Qualitative data collection and analysis

In the qualitative analysis, stakeholders involved in the ongoing implementation of VBHC for one year beginning in March 2014 were interviewed. We interviewed five stakeholders (non-clinicians and clinicians) who had a strategic role in the implementation of VBHC, and 14 stakeholders (non-clinicians and clinicians) who worked hands-on with the implementation at the hospital. The majority of health-care professionals (80%) had combined clinician/managerial positions.

Semi-structured interviews were performed to capture interviewees’ perceptions of value with focus on outcome vs cost (the value quota), focusing on patients’ perception of value and the ability to capture individual patient costs in the organisation. Primarily open-ended questions were posed. All interviewees received both written and oral information about the research project and gave their consent to participate in the study. All interviews were tape-recorded (duration from 27–70 min) and transcribed verbatim. Two from the interdisciplinary research group performed the initial analysis by reading the transcripts several times. Qualitative content analysis (Graneheim and Lundman, 2004) was used to structure and analyse the text. Graneheim and Lundman's (2004) content analysis method is a structured approach in qualitative research, starting with the identification of “meaning units” in the text, which are clusters of words, sentences or paragraphs with a central meaning related to the study’s objectives. These meaning units are then condensed, shortening the text while preserving its core content to make the data more manageable. Following this, the condensed units are coded, assigning labels or categories that reflect their content and context. These codes are grouped into subcategories based on similarities, and then into broader categories, representing a higher level of abstraction. The final step is the development of categories/themes, which are threads of underlying meaning that tie together the condensed units, codes, subcategories and categories, ultimately providing a narrative that reflects the essence of the participants’ experiences and perceptions in relation to the research question. This process ensures that the analysis remains grounded in the data, allowing for meaningful interpretation and a credible understanding of the research topic. In the first naïve coding, we used inductive reasoning to analyse the text, focusing our approach on the perception of value and the relationship between outcome and cost as the creators of value. After the naïve coding, categories were created to further structure the data related to value perception. In the second stage, a more deductive approach was chosen where we used the concept of professional logic to find differences between interviewees with a clinical or non-clinical background to investigate differences and similarities between different institutional logics with regard to the perception of value. The manifested and latent messages were discussed and revised among the authors to reach an agreement about coding and the formulation of the themes. The qualitative analysis was structured using NVIVO 9 software, QRS International.

The study was conforming to the ethical standards as expressed by the World Medical Association’s (2013) Declaration of Helsinki. Participants were provided with detailed information about the study's both via email and phone. Ethical demands for qualitative research, informed consent, confidentiality, the consequences of the study and role of the researchers, have been considered and followed.

## Results

In total, the study comprised 19 interviewees, as displayed in Table 1, divided into clinicians and non-clinicians, as they may have been driven by separate professional logics (see Table 2). Two main categories emerged: the different logic’s impact on value and the challenge of working with cost.

### The different logic’s impact on value

Interviewees perceived VBHC as a helpful tool to increase the value delivered to individual patients. However, there were differences between clinicians and non-clinicians regarding the perception of value. Clinicians frequently appreciated VBHC, as they perceived its introduction as an opportunity to focus more on outcomes for patients and less on cost containment. In contrast, non-clinicians talked about VBHC in terms of a strategic framework for governance of health-care organisations, or as a framework for closely monitoring different continuous improvement processes. When asked specifically about what value meant to them, the interviewees focused primarily on how outcomes were talked about within their own professional group. It is notable that many of the clinicians did not explicitly mention costs, one of the central aspects of the VBHC value quota. Non-clinicians perceived outcomes as the key component driving value, but frequently described value as related to costs, and occasionally, explicitly described value as related to efficiency, as intended by the concept of VBHC. Some interviewees indicated that the introduction of VBHC contributed to an increased focus on clinical outcomes at all levels of the hospital hierarchy, suggesting a shift in institutional logic at the studied hospital from the managerial control logic of care production (volume and flow) towards more focus on medical outcomes and the professional cure and care logic.

### The challenge of working with cost

Most interviewees perceived costs per care episode as difficult to track accurately. The current accounting systems did not support this at the studied hospital. Securing the full cost to all involved health-care providers over the full care cycle was perceived as desirable by interviewees, but virtually impossible to accomplish. Non-clinicians and clinicians used different terms when describing the costs. Some referred to cost as an “investment” while others considered it an “expense”. Some interviewees, particularly clinicians, expressed the opinion that investments to improve outcomes and efficiency were impossible (e.g. hiring more physicians able to see patients) if the improvement increased cost.

The lack of exact per-patient cost data forced the project groups implementing VBHC at the case hospital to work with different proxy for cost. They focused on the activities demanding the most resources, that is, cost drivers, such as length of hospital stay or number of follow-up visits.

## Discussion

While value is a central concept in VBHC, the perception of value was different among the stakeholder groups at the studied hospital. The majority of respondents did not appear to focus much on costs or cost containment, even though that objective is as potent as improving outcomes for enhancing the value quota within the VBHC framework. Clinicians talked about value as a dynamic concept depending on the view of the patient and the context, and put forward the fact that better outcomes sometimes require higher cost or more resources. While that clinical perception surely makes sense, it was evident that clinicians did not fully understand the value quota and more often talked about improving outcomes, rather than reducing costs. It might be interesting to note that the concept of VBHC entitles you to increase the cost, if the value quota is improving. In other words, as long as the increase in outcome value, as perceived by the patient, is larger than the additional cost for achieving such value, an increase in resources is possible (Porter and Teisberg, 2006, p. 98).

In contrast, non-clinicians’ perception of value appeared to be more aligned with a cost-controlling or at least a cost-focused perspective. While many interviewees indicated that the deficiencies of the current accounting systems hamper the understanding of the cost, the results show that there is a discrepancy between the concepts of value between clinicians and non-clinicians.

At the same time, there were experiences presented in the results of how VBHC was experienced as a shift from the managerial control logic towards more of the professional cure and care logic. This is in line with the notion of hybrid organisations, blending management logics with clinical logics, as found by Ramsdal and Bjorkquist (2019) in another VBHC implementation.

The challenge of working with cost was clearly articulated. The hospital accounting systems were brought forward as creating a virtually impossible situation to capture the cost per care episode. Other research studying value-based implementation work, has found how, improved coordination among health professionals work contributed to increased cost-effectiveness (Miettinen and Tenhunen, 2020). The uncertainty introduced by the ambiguous term “value”, may present an obstacle to organisations attempting to manage a shift in how they operate, as the ambiguity may introduce friction between professional groups, management and between patients and providers. Paradoxically, this uncertainty around what value means could also be a leadership opportunity and a way of integrating the different perceptions among multiple stakeholders. By use of previous research, we suggest that the managerial model of distributed leadership, with a focus on increased influence via co-creation of involved actors, the understanding of different needs and conditions and integration via interpersonal interactions rather than formal roles and responsibilities, provides a fertile ground to further VBHC (Avolio *et al.*, 2009; Currie and Lockett, 2011; Harris and DeFlaminis, 2016). In light of this, creating a shared understanding of the value concept among the involved stakeholders may be pivotal to enhance future implementation processes of VBHC.

The different perceptions of the term “value” as found in this study are potentially connected to the different meanings in many leadership terms and the potential dichotomy between “management vs leadership”. While this empirically informed study will not be dissecting the specific terms, we like to suggest that regardless of the terminological definitions, in practice the key challenge is about balancing clinical bedside needs with economical constraints. Bååthe *et al.* (2022) argued health-care top managers need to be balancing quality of patient care, economy and professionals’ engagement to increase sustainability in health care. They further suggest how that balancing act is not an anomaly top managers can eradicate. Instead, managers should deliberately act with a notion of continuous balancing in mind, which can create virtuous development spirals where managers and health professionals closely collaborate. Hence, co-creation by distributed leadership may be of great importance for sustaining organisational development (Avolio *et al.*, 2009; Currie *et al.*, 2011; Spillane, 2005; Harris and DeFlaminis, 2016). It is further suggested that when co-creative practices emerge in a constructive and trusting way, the relation between managers and health professionals can be developed into a joint agency, benefitting all health-care development (Raelin, 2016).

At the same time, a study by Nilsson and Sandoff (2017) shows the importance of goal setting, role description and leadership expectations while implementing VBHC, which in previous implementation research has been described as a contextual dialogue with the aim of integrating different ways of understanding (Bååthe and Erik, 2013). Bååthe and Norbäck present how both the managerial and the professionals’ understanding need to and can be modified, and they outline how that contextual dialogue can happen as a conversation-based collaborative perspective. It provides an alternative to the prevailing managerial control perspective and they further suggest the initiator responsibility lies firmly at the managerial level (ibid). It was clear from the empirical result how clinicians and non-clinicians had different perspectives, and research has been in agreement that it should not be taken for granted that people involved in a change process share the same goals, commitment or understanding of the concept being implemented (Schein, 1993; Loup and Koller, 2005). Storkholm *et al.* (2017) further this line of reasoning and present a successful change story integrating the managerial and the professional needs and wants. It shows that when leadership deliberately takes the position of the clinical professionals when initiating a change process, and when focusing on improving patient quality in the care process (instead of focusing on economy), engagement from clinicians is likely to follow. Storkholm *et al.* clarify how also economical results are improved, but now as a positive side effect, from working with enabling patient care to be delivered in a more efficient and effective way.

While the patient in VBHC is described by Porter as acting as a “consumer” who chooses the best provider based on outcome or cost, we would suggest there are no consumers of health care, just individuals seeking medical, nursing or other health-care services. The logic of the patient may not be transferable to the underlying assumption of a customer being both able and willing to compare value for money, that is, price–performance ratios provided by different health-care providers. From a European perspective, there is insufficient evidence that patient choice as a competitive driver in health care has improved outcomes or reduced cost (Anell *et al.*, 2012; Okma and Crivelli, 2013; Siciliani *et al.*, 2022). At the same time, the modern patient is more than a passive recipient of care, or a consumer of service alone; rather, the patient should be acknowledged as a partner in care, co-creating value together (Coulter *et al.*, 2015; Britten *et al.*, 2020).

Traditionally, the logic of medicine, which is driven by improving outcomes and treatment efficacy, is considered the most influential and powerful. This could impact the chances of other logics to be heard at both the strategic and operational levels of VBHC; for example, the logic of care (such as nursing) or the logic of control (such as managers). We, therefore, argue that the implementation of VBHC needs to actively work towards balancing stakeholders’ multiple needs and integrate different logics, while also providing dedicated space to further the patients’ voice in this change process (Nilsson *et al.*, 2017). While the voices of the clinicians and the non-clinicians are important, it could be argued that the voice of the patient must be further listened to develop a sustainable health-care model that is more efficient and effective while at the same time becoming more person-centred (Britten *et al.*, 2016; Coulter *et al.*, 2015).

## Limitations and suggestions for future research

While the scope of VBHC is holistic and includes all levels of health care, including patients’ residences and primary care centres, this study was limited in scope to the hospital setting.

The collected data were primarily limited to the implementation of VBHC at one hospital, so while it may not be possible to extrapolate the findings, one should remember that in qualitative research, unlike in quantitative research, the goal is not generalisation in the statistical sense. Qualitative methodology does not produce findings that claim general applicability irrespective of context. Instead, there is a deliberate focus on the contextual specificity. However, as suggested by Malterud (2001, 2014), this focus on context does not preclude transferability. Van Maanen and Barley (1984) found significant commonalities in health-care contexts across the Western world and Larsson (2009) suggests that the usage of a piece of research is a dynamic act, which is completed if, and only if, someone else can make sense of situations or processes or other phenomena with the help of descriptions from the research texts.

Another limitation is that our data represents a data set that followed an implementation process that occurred almost ten years ago. The discontinuation of VBHC in Sweden in 2017 was primarily due to a scandal involving unethical practices between a consultancy group and a top university hospital. This significantly tarnished VBHC’s reputation and halted its progress nationwide (Krohwinkel *et al.*, 2019). Despite this setback in Sweden, VBHC remains a topic of interest and implementation globally, with the Swedish experience offering valuable lessons for other countries. Our study aims to address the knowledge gap identified in the 2022 scoping review on VBHC by van Staalduinen *et al.* (2022), focusing on implementation challenges and strategies. By sharing our insights, we contribute to the advancement of VBHC discussions and practices worldwide. We echo the review’s call for an interdisciplinary approach, combining health-care insights with broader management research to enrich the VBHC dialogue and development.

Qualitative research, including interviews, inherently involves a degree of subjectivity. The interpretation of qualitative data can be influenced by the researcher’s perspectives, biases and the specific context in which the data were collected. The interviewees in this study were predominantly clinicians in managerial positions. This selective sampling could lead to a biased perspective, as these individuals may have different experiences, viewpoints and insights compared with other health-care professionals, particularly those in non-managerial roles or different health-care settings. Qualitative research often grapples with such selection biases, and by being in an interdisciplinary and multi-professional research group we have worked towards balancing the risk of one voice or one profession overpowering the others. A significant limitation of this study is the lack of qualitative data from patients. Patients are key stakeholders in VBHC, and while this study had its focus on the hospital employees, the exclusion of patient voices should be acknowledged, as it can lead to an incomplete understanding of VBHC implementation challenges.

Future research in VBHC should prioritise including patient perspectives to understand how the value ratio evolves over time with the implementation of value-based health care practices. This exploration should examine whether improvements in the value ratio result from enhancing the clinical outcomes that patients value (the numerator), increasing the efficiency by reducing the costs of delivering these outcomes (the denominator) or a combination of both. Importantly, research should also consider studies where patients are actively involved in educating hospital staff about the outcomes they value most. This participatory approach could lead to co-creative development processes aimed at enhancing the value ratio, taking into account that increasing value may also involve accepting higher costs when it leads to outcomes that patients deem significantly valuable.

## Conclusions

Our findings indicate that there are large variations in how different hospital stakeholders perceive the concept of value when implementing VBHC.

In the present study, the clinicians, as primarily propagated by the professional group of physicians, appear to have a dominant influence on this logic. Improving clinical outcomes was the most important way to improve the value of health care provided. This was done by prioritising outcomes. Improvement of outcomes was prioritised over cost containment or cost reduction.

If the concept of value within VBHC is driven by clinicians’ traditional assumptions of what value means to the patients, there is a risk that history will simply be repeated and the international goal for more person-centred care innovation will not be met. While clinicians’ voices are important, a multitude of voices must be integrated, including patients’ and non-clinicians’, for VBHC to become a sustainable care model and deliver on the promise to allow the patients’ voices to be heard while increasing health-care efficiency and effectiveness. Health-care leaders need to ensure the patients’ and the non-clinicians’ perceptions of value are integrated and balanced with the clinical perception if VBHC is to deliver on its promise.

## Figures and Tables

**Table 1.** Stakeholder interviews

Profession	Strategic level	Operative implementation	Sum
	Non-clinicians		
Management consultants	2		2
Financial controllers		3	3
Logistician		1	1
	Clinicians		
Registered nurse	1		1
Physician	1	8	9
Pharmacist	1		1
Psychologist		1	1
Occupational therapist		1	1
Sum	5	14	19

**Source:** Authors’ own work

**Table 2.** Examples of perceptions of outcomes, costs and value among clinicians and non-clinicians

Clinicians	Non-clinicians
*The institutional logic’s impact on value*	
*We work so much more with outcomes than with costs. That is the brutal truth*	*If you try to get the health-care professionals to produce more, then you will not get much attention or they won’t agree with you. But if you have a clear target for what value you want to achieve, you have a higher chance of success.*
*At the hospital, of course we see the cost and how it impacts our operation. But, of course, there is a cost to the patient and society that we do not acknowledge, and if you want to deliver value to the patient, you need to see the entire picture*	*You can’t separate clinical outcomes and cost. If you work with the numerator (outcome) then you should let the denominator (cost) be unchanged, and vice versa*
*For me, value is something personal; I think you should ask the patient so they can express what’s important to them, and that differs, of course, depending on their condition, age, education and so forth, so it’s important to get the patients’ perspective*	*The objective is to deliver as much health care as possible for every Swedish crown spent, as there are limited resources available to deliver quality care to the patient using less or the same measure of resource*
*The challenge of working with cost*	
*… we haven’t done that, only the economists know what the cost is*	*We don’t measure the patient pathway or the cost per patient. Although, in this case that would be preferable. So, the systems are not tuned to this way of working…*
*The concept of cost is extremely difficult. It isn’t that outcomes are easier, but they are easier to understand, more like a process measurement*	*Because we lack a perfect per patient cost system, we use a resource-based system instead. In many cases, the length of the hospital stay is the single most important cost driver, so it’s a good proxy for cost*

**Source:** Authors’ own work
